# The Effect of Sputtering Sequence Engineering in Superlattice-like Sb-Rich-Based Phase Change Materials

**DOI:** 10.3390/ma17112773

**Published:** 2024-06-06

**Authors:** Anding Li, Ruirui Liu, Liu Liu, Yukun Chen, Xiao Zhou

**Affiliations:** 1 School of Materials Science and Engineering, Shanghai Institute of Technology, Shanghai 201418, China; a2476157229@163.com (A.L.); liuliu020623@163.com (L.L.); growl_joke@163.com (Y.C.); 2 State Key Lab of Metal Matrix Composites, School of Materials Science and Engineering, Shanghai Jiao Tong University, Shanghai 200240, China

**Keywords:** phase change materials, superlattice-like thin film, sputtering sequence, phase change properties, thermal stability

## Abstract

This paper presents a comprehensive investigation into the thermal stability of superlattice-like (SLL) thin films fabricated by varying the sputtering sequences of the SLL [Ge_8_Sb_92_(25nm)/GeTe(25nm)]_1_ and SLL [GeTe(25nm)/Ge_8_Sb_92_(25nm)]_1_ configurations. Our results reveal significantly enhanced ten-year data retention (*T_ten_*) for both thin films measured at 124.3 °C and 151.9 °C, respectively. These values surpass the *T_ten_* of Ge_2_Sb_2_Te_5_ (85 °C), clearly demonstrating the superior thermal stability of the studied SLL configurations. Interestingly, we also observe a distinct difference in the thermal stability between the two SLL configurations. The superior thermal stability of SLL [GeTe(25nm)/Ge_8_Sb_92_(25nm)]_1_ is attributed to the diffusion of the Sb precipitated phase from Ge_8_Sb_92_ to GeTe. This diffusion process effectively reduces the impact of the Sb phase on the thermal stability of the thin film. In contrast, in the case of SLL [Ge_8_Sb_92_(25nm)/GeTe(25nm)]_1_, the presence of the Sb precipitated phase in Ge_8_Sb_92_ facilitates the crystallization of GeTe, leading to reduced thermal stability. These findings underscore the significant influence of the sputtering sequence on the atomic behavior and thermal properties of superlattice-like phase change materials. Such insights provide a robust foundation for the design and exploration of novel phase change materials with improved thermal performance.

## 1. Introduction

With the swift advancement of 5G, artificial intelligence, and related technologies, humanity has ushered in the epoch of big data. Concomitantly, these remarkable progressions have imposed exacting requirements on memory, encompassing storage density, power consumption, and switching rate. Phase change random access memory (PCRAM) has garnered considerable attention owing to its exceptional scalability, data retention, storage density, and compatibility with complementary metal oxide semiconductor (CMOS) technology [1]. Hence, it is widely acknowledged as a formidable candidate for the upcoming generation of non-volatile memory. PCRAM primarily leverages the high resistance of the amorphous state of the chalcogenide compound and the low resistance of the polycrystalline state to effectively store logical state information. The electric pulse-induced heat facilitates the conversion of the phase change materials (PCMs) between the high resistance state (amorphous state) and the low resistance state (crystalline state), effectively utilizing the resistance variation for the data storage of “0”and “1” [2,3,4,5].

The properties of PCMs are pivotal in determining the performance of PCRAM. Currently, the focus has predominantly centered on the relatively mature PCMs with the Ge-Sb-Te composition system, including Ge_2_Sb_2_Te_5_, Ge_1_SbTe_4_, and Ge_1_Sb_4_Te_7_ [6,7,8]. However, the limited thermal stability of Ge-Sb-Te (GST) has hindered its extensive commercial application [9,10,11]. Consequently, the pursuit of PCMs that exhibit both rapid phase change speed and high thermal stability has emerged as a prominent and active research area in recent years.

However, phase change speed and thermal stability represent a pair of parameters with an inverse relationship, including that an increase in phase change speed is concomitant with a decrease in thermal stability and vice versa. In this context, TC Chong introduced the concept of the “superlattice-like (SLL)” in PCMs [12] to strike a balance between these characteristics. Specifically, the superlattice is formed by alternating layers of two or more different materials on a nanometer scale. By precisely controlling the thickness and order of each layer, the physical and chemical properties of the material can be significantly altered. The properties of PCMs can be modulated by the alternate deposition of two PCMs with distinct characteristics, one possessing a higher crystallization rate and the other exhibiting greater thermal stability. Moreover, SLL demonstrates a reduced programming current and enhanced operation speed, resulting in lower power consumption while also enabling multi-stage phase transition. The previous works have demonstrated that PCMs with a superlattice-like structure, such as SnSb_4_/ZnSb [13],Ge_8_Sb_92_/Ga_30_Sb_70_ [14], and Ge_8_Sb_92_/Ge [15], exhibit superior switching speed and reduced energy consumption during phase transition. However, the effect of the sputtering sequence on the phase transition characteristics of SLL thin films remains unexplored, and a comprehensive design principle for SLL thin films necessitates further investigation.

It has been reported that the crystallization mechanism of PCMs is intricately linked to their phase transition. Specifically, it has been observed that growth-dominant crystallization represents a faster process compared to nucleation-dominant crystallization [16,17]. The majority of Sb-based PCMs, exemplified by Ge-Sb [18], predominantly exhibit a growth-dominant crystallization mechanism, resulting in their fast phase change speed. However, despite this advantageous characteristic, the limited thermal stability of Sb-based PCMs has impeded the progress of the scientific research in this domain. In contrast, Ge-Te displays a rapid phase change speed owing to similar growth-dominant crystallization mechanisms. Additionally, Ge-Te exhibits high thermal stability and a substantial resistance ratio between the amorphous and crystalline states [19]. Consequently, Ge-Te is considered as a promising PCM in the field of PCRAM. Wu et al. [20] have reported that SLL Ge_8_Sb_92_/GeTe shows high-density and high-speed phase transition characteristics. Our earlier studies have validated the presence of an Sb precipitation phase in Sb-rich PCMs when annealing at low temperatures, even at the deposited state. Concurrently, the Sb precipitation phase significantly impacts the phase change properties of Sb-rich-based SLL PCMs [21]. Hence, it can be inferred that the sputtering sequence may also influence the properties of Sb-rich-based SLL PCMs to a certain extent. However, there remains a lack of sufficient correlated research in this topic [19].

In this research, we have produced various types of SLL-GeTe-Ge_8_Sb_92_ configurations with varying periods. For the sake of simplicity, we focus primarily on the SLL-GeTe- Ge_8_Sb_92_ with a single period as our main research system. We systematically analyze the phase change properties of the SLL structures, namely SLL [GeTe(25nm)/Ge_8_Sb_92_(25nm)]_1_ and SLL [Ge_8_Sb_92_(25nm)/GeTe(25nm)]_1_, which feature different sputtering sequences. Our findings reveal that different sputtering sequences imply distinct crystallization mechanisms in SLL-GeTe-Ge_8_Sb_92_, providing a basis for advancing the development of PCMs with improved phase change properties.

## 2. Experimental Procedure

### 2.1. Sample Preparation

SLL-GeTe-Ge_8_Sb_92_ were prepared by alternating sputtering of Ge_8_Sb_92_ and GeTe alloy targets (99.99 at.%) on Si/SiO_2_ (100) substrates. Substrate consists of a silicon base with an additional 3000 Å layer of SiO_2_. The equipment used in the experiments is a multi-target magnetron sputtering system, model GP450, manufactured by Shenyang Tengao Machinery Manufacturing Co., Ltd. (Shenyang, China). The single-period [Ge_8_Sb_92_ (25nm)/GeTe (25nm)]_1_ and [GeTe(25nm)/Ge_8_Sb_92_(25nm)]_1_ superlattice-like thin films are the main focus of our research and can be abbreviated as SLL GS/GT and SLL GT/GS. The abbreviation SLL GS/GT ([Ge_8_Sb_92_ (25nm)/GeTe (25nm)]_1_) denotes the deposition sequence of Ge_8_Sb_92_ (GS) followed by GeTe (GT), while SLL GT/GS ([GeTe(25nm)/Ge_8_Sb_92_(25nm)]_1_) represents the sequence of GeTe (GT) followed by Ge_8_Sb_92_ (GS). The thickness of the thin film is directly controlled by the deposition time, and the final thickness is about 50 nm. For different target materials, we sputter for 30 min under identical experimental conditions and measure the thickness using a step profiler. By correlating the sputtering time with the measured thickness, we determine the sputtering rate for each target material. When preparing samples for experiments, we achieve the desired thickness by controlling the sputtering time accordingly. Based on experimental measurements, the sputtering rates for the Ge8Sb92 target and the GeTe target are 1.25 nm/s and 0.43 nm/s, respectively. The multi-period thin films signify variations in each thin film thickness, i.e., [Ge_8_Sb_92_(6nm)/GeTe(6nm)]_4_, [GeTe(6nm)/Ge_8_Sb_92_(6nm)]_4_, [Ge_8_Sb_92_(5nm)/GeTe(5nm)]_5_, and [GeTe(5nm)/Ge_8_Sb_92_(5nm)]_5_ (see the Supplementary Materials for details). The sputtering background vacuum is better than 2 × 10^−4^ Pa, with a sputtering power of 20 W. Deposition took place in an Ar atmosphere at a flow rate of 30 sccm and a pressure of 0.2 Pa. The substrate is rotated at a rotation speed of 20 rpm to ensure deposition uniformity.

### 2.2. Material Characterization

A custom-made two-point-probe setup was utilized to measure the in situ temperature-dependent resistances (*R*-*T*) of the thin films in the Ar atmosphere, and the same technique was employed for processing the annealing samples. The resistance of the thin film is measured by contacting two metal probes from the testing system with the surface of the film sample. By observing the variation in resistance with temperature, we can determine the corresponding transition temperature ($T_{c}$) of the phase change materials. The heating platform temperature is regulated by the TP94 temperature control system from Linkam Scientific Instruments Ltd., (Warrington, UK). Cooling is achieved through the LNP94/2 cooling system, which utilizes liquid nitrogen for control. The maximum heating rate of this system can reach 90 °C/min. The resistance of the samples was assessed at multiple heating rates, and the crystallization activation energy was determined utilizing the non-isothermal Kissinger formula. The 10-year data retention of the investigated thin films was ascertained by employing the Arrhenius equation, based on isothermal changes in resistance at elevated temperatures. The amorphous and crystalline structures of the superlattice-like thin films were characterized using Raman spectroscopy at 785 nm; the excitation source is an Ar ion laser with a frequency resolution of 1.5 cm^−1^. The crystal phases of the thin films before and after annealing were observed by X-ray diffraction (XRD), with a 2θ range of 20–60° and a scanning rate of 2°. The equipment used in this study is a Rigaku D/max 2550VB3+/PC fully automated XRD system, equipped with an 18 kW rotating anode generator. It has a resolution of 0.002° (2θ) and uses a copper target as the X-ray source with a corresponding wavelength of 1.54056 Å (Cu Kα). The device was procured from Bruker Corporation in Berlin, Germany. The operating voltage and current are 40 kV and 100 mA, respectively. The surface morphology and roughness were evaluated by atomic force microscopy (AFM), model MFP-3D.

## 3. Results and Discussion

**Figure 1:**
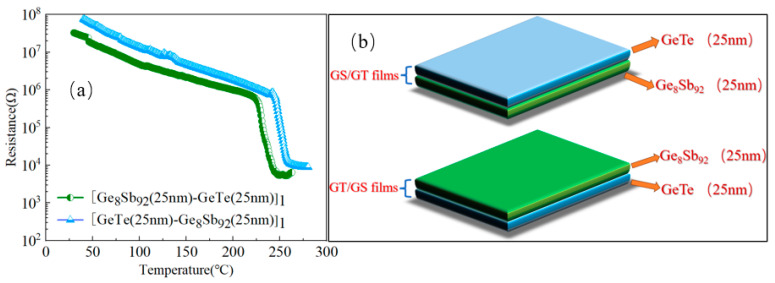
(**a**) Temperature-dependent sheet resistance curves of SLL GS/GT and SLL GT/GS at a heating rate of 10 °C/min. (**b**) The structural schematic of SLL GS/GT and SLL GT/GS.

Figure 1a presents the temperature-dependent sheet resistance curves (*R*-*T*) of SLL GS/GT and SLL GT/GS, acquired at a heating rate of 10 °C/min. Simultaneously, the *R*-*T* curves for the remaining SLL-GS-GT with multiple periods can be found in Figure S1 (refer to the Supplementary Materials for additional information). Additionally, Figure 1b displays their respective structural schematic diagrams. In Figure 1a, we observe a gradual reduction in resistance with increasing temperature in the initial stages. This intriguing phenomenon can be attributed to the semiconductor characteristics exhibited by the deposited thin film [22]. As the temperature continues to rise, a remarkable cliff-like decrease in the resistance emerges in the thin films. This striking behavior can be primarily attributed to the transformation of the thin film from an amorphous state to a crystalline state. The temperature corresponding to this sharp resistance decrease defines the crystallization temperature (*T_c_*). Subsequently, as the temperature further increases, the resistance reaches a stable plateau, indicating the establishment of a thermodynamically steady state. In our investigation, the *T_c_* values of SLL GS/GT and SLL GT/GS are 228.3 °C and 250.3 °C, respectively, both surpassing the *T_c_* of GST (approximately 150 °C). Notably, SLL GT/GS exhibits a higher *T_c_* value compared to SLL GS/GT, including under multiple cycles, indicating the superior thermal stability in the amorphous state of SLL GT/GS [23]. Moreover, a disparity in the resistance between SLL GS/GT and SLL GT/GS is revealed in both their amorphous and crystalline states. SLL GT/GS is demonstrated to have a higher resistance level compared to SLL GS/GT, rendering it an attractive candidate for reducing the power consumption in PCRAM applications.

**Figure 2:**
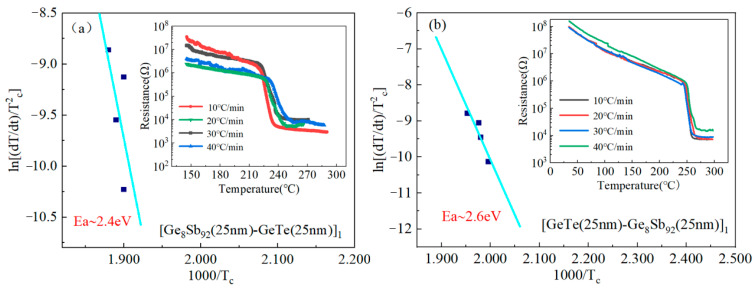
The Kissinger plots in $\left[(dT/dt)/T_{c}^{2}\right]$ versus 1/kT of SLL GS/GT (**a**) and SLL GT/GS (**b**) thin films. Illustration includes the temperature-dependent thin-layer resistance curves of SLL GS/GT and SLL GT/GS thin films with different heating rates.

Crystallization activation energy (*E_a_*) is a pivotal parameter for assessing the thermal stability of PCMs. Kissinger’s original method involved the kinetics of chemical reactions. Subsequently, this method has been developed and also used for the crystallization of annealed amorphous materials. To obtain this critical value, the non-isothermal crystallization kinetics Kissinger method is applied. The specific formula for calculating *E_a_* is as follows [24]:

$$\ln[(dT/dt)/T_{c}^{2}]=C+E_{a}/(k_{b}T_{c})$$

where dT/dt is the heating rate in the crystallization process (*dT*/*dt* = 10 °C/min, 20 °C/min, 30 °C/min, and 40 °C/min), $T_{c}$ is the phase change temperature, and the $T_{c}$ values for SLL GS/GT are determined as 228.3 °C (10 °C/min), 229.7 °C (20 °C/min), 230.3 °C (30 °C/min), and 236.7 °C (40 °C/min); and those for SLL GT/GS are 250.3 °C (10 °C/min), 250.9 °C (10 °C/min), 251.4 °C (10 °C/min), and 253.6 °C (10 °C/min), as shown in the inset of Figure 2. *C* represents a constant, $E_{a}$ stands for crystallization activation energy, and $k_{b}$ denotes the Boltzmann constant with a specific value of 8.617 × 10^−5^ eV K^−1^. Figure 2 illustrates the Kissinger curves, plotting $ln[(dT/dt)/T_{c}^{2}]$ against 1/kT for SLL GS/GT and SLL GT/GS. The results show that their *E_a_* values are 2.4 and 2.6 eV, respectively, which are higher than that of GST (~2.28 eV). The larger *E_a_* indicates better thermal stability, which in turn ensures reliable memory programming [16].

**Figure 3:**
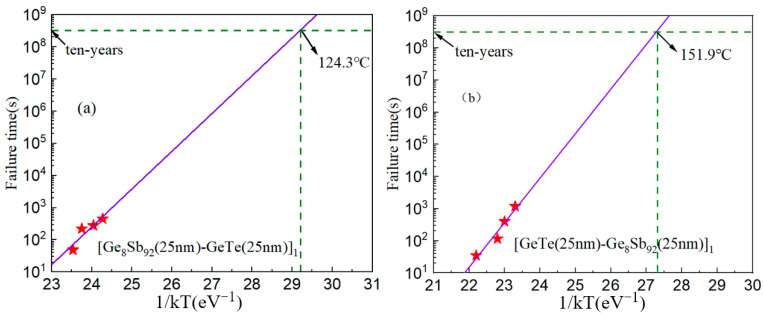
Failure time versus reciprocal temperature of SLL GS/GT (**a**) and SLL GT/GS (**b**).

Moreover, understanding the ten-year data retention (*T_ten_*) of PCMs also offers valuable insights into their thermal behavior. *T_ten_* represents the temperature at which a PCM can be applied for a period of 10 years. This parameter can be obtained by the isothermal molecular dynamics simulation method based on the Arrhenius equation [19]:

$$t=\tau_{0}\exp(\frac{E_{a}}{k_{b}T})$$

where t is the failure time, which is defined as the time to retain 50% of the resistance. *τ*_0_ is a pre-exponential factor depending on the properties of the material. $E_{a}$ is the crystallization activation energy. $k_{b}$ is Boltzmann constant, and T is the isothermal annealing temperature of the thin film [16]. As shown in Figure 3, the T*_ten_* values of SLL GS/GT and SLL GT/GS are 124.3 °C and 151.9 °C, which are much higher than that of GST (~85 °C) [23]. The notably high *T_ten_* values of SLL GS/GT and SLL GT/GS indicate their remarkable thermal stability. Additionally, their superior thermal stability can meet the stringent requirements of automotive electronics applications, which typically demand reliable operation at an elevated temperature of approximately 120 °C [25].

**Figure 4:**
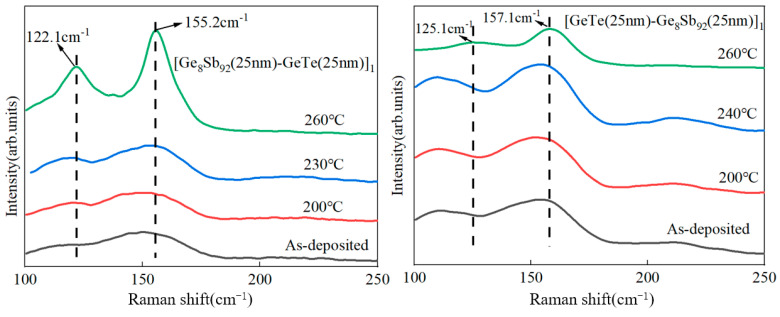
Raman spectra of SLL GS/GT (**left**) and GT/GS (**right**) at various annealing temperatures for 3 min.

Figure 4 presents the Raman scattering spectra of SLL GS/GT and SLL GT/GS in their as-deposited state and subsequent annealing at different temperatures. The Raman spectra of the as-deposited thin films exhibit a broad “steamed bread-like” peak, indicative of a disordered atomic structure and the amorphous state of the thin films [26,27,28]. As the annealing temperature increases, the Raman spectra gradually transform from the disordered broad peak to relatively sharp Raman peaks, signaling the progressive crystallization of the thin films. Two distinctive Raman peaks are observed at approximately 122 cm^−1^ and 155 cm^−1^. The peak at 122 cm^−1^ corresponds to the A1 vibration mode of the corner-sharing tetrahedral unit GeTe_4-n_Ge_n_ [29], while the peak at 155 cm^−1^ is attributed to the Sb Raman scattering peak mode of the typical A7 phase [30]. With further increments in the annealing temperature, the Raman spectrum peaks become sharper, indicating the gradual growth of the crystallization grain and an increased degree of crystallization [31]. Furthermore, an increase in the annealing temperature leads to a blue shift in the Raman peak. This blue shift is believed to be caused by the alteration of the internal stress following the complete crystallization of the two layers. The finding implies that the interatomic force is stronger in long-range or staggered crystals, resulting in a wider band gap due to the quantum confinement effect [32].

Upon the complete crystallization of SLL GT/GS, a notable decrease in the Raman peak intensity at 125.1 cm^−1^ is observed. Our hypothesis suggests that this reduction is attributed to the diffusion of the Sb phase from GS into the GT layer. As a result of this diffusion, some Ge and Te atoms are replaced by Sb atoms, leading to a reduction in the tetrahedral structural units in GeTe_4-n_Ge_n_ (n = 0, 1, 2, 3, and 4) and Sb–Sb bonds. Furthermore, the diffusion of Sb also mitigates the impact of the Sb precipitation on the thermal stability of the thin film, resulting in the higher thermal stability observed in SLL GT/GS compared to SLL GS/GT. This observation provides an explanation for the observed difference in *T_c_* between SLL GS/GT and SLL GT/GS, as illustrated in Figure 1.

**Figure 5:**
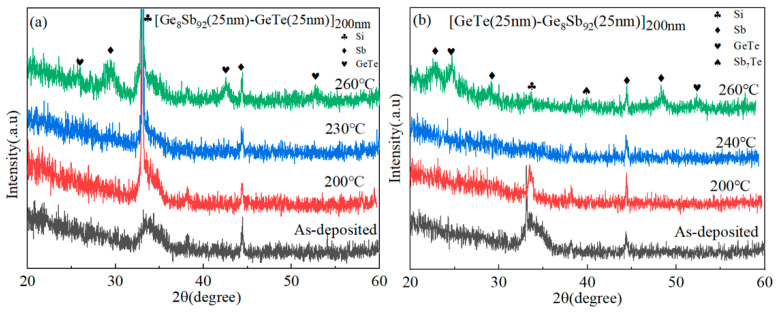
XRD plots of SLL GS/GT (**a**) and SLL GT/GS (**b**) at various annealing temperatures for 3 min.

Figure 5 displays the X-ray diffraction (XRD) patterns of the as-deposited and annealed SLL GS/GT and SLL GT/GS thin films. Notably, both XRD patterns reveal the presence of a small Sb diffraction peak, indicating the precipitation of the Sb phase in the as-deposited state. It has been reported that the presence of a small amount of an Sb precipitated phase can enhance the phase change speed of the thin film [33]. Following complete crystallization, a significant increase in the intensity of the diffraction peak is observed, indicative of the improved crystallinity in the thin films. Interestingly, distinct differences are observed in the diffraction peaks of SLL GS/GT and SLL GT/GS after complete crystallization. This disparity can be primarily attributed to the change in the preferred orientation of the Sb and GeTe crystal planes, potentially influenced by the interfaces of SLL structures with different sputtering orders. Furthermore, the crystallized SLL GT/GS exhibits a distinct diffraction peak corresponding to Sb_7_Te, which is in line with the conclusion derived from the Raman spectra presented in Figure 4.

**Figure 6:**
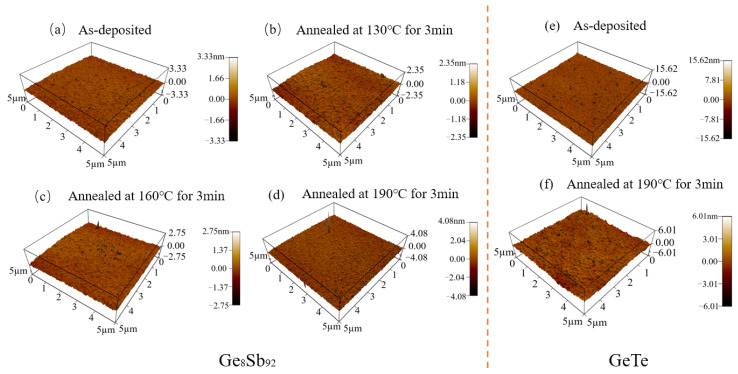
Three-dimensional AFM topographic images of Ge8Sb92: (**a**) As-deposited; (**b**) Annealed at 130 °C for 3 min; (**c**) Annealed at 160 °C for 3 min; (**d**) Annealed at 190 °C for 3 min. Three-dimensional AFM topographic images of GeTe: (**e**) As-deposited; (**f**) Annealed at 190 °C for 3 min.

In order to investigate the disparity in the thermal stability between SLL GS/GT and SLL GT/GS, we performed AFM to analyze the surface properties of GT before and after annealing, as well as GS at different temperatures. Figure 6 depicts the surface topography of GT and GS. In their as-deposited state, GS exhibits a surface roughness of 2.462 nm, whereas GT has a surface roughness of 0.846 nm. After annealing at 130 °C and 160 °C, the surface roughness of GS increases to 1.248 nm and 1.296 nm, respectively. Upon complete crystallization at 190 °C, the surface roughness of GS significantly increases to 6.816 nm, while GT exhibits a surface roughness of 5.617 nm. This substantial disparity in the surface roughness between GS and GT before and after crystallization highlights the notable difference in the phase transition behavior between them.

The primary distinction between SLL GS/GT and SLL GT/GS lies in their sputtering sequences, resulting in different thermal orders for the multilayer thin films. In the case of SLL GT/GS, the GT layer serves as the underlayer with high thermal stability. The diffusion of the Sb precipitated phase into the GeTe layer, coupled with the suppression of the Sb phase precipitation, contributes to the enhanced thermal stability of SLL GS/GT. In comparison, for SLL GS/GT, GS is positioned as the lower layer, making it more susceptible to heat promotion, leading to the precipitation of the Sb phase and an increase in the surface roughness. Pieterson et al. [34] have reported that the crystallization initiates at the crystalline–amorphous interface. The Sb phase precipitated at the GS interface favors the formation of a crystalline–amorphous interface between Sb (crystalline) and GT (amorphous), thereby promoting the crystallization of GT. This distinct crystallization mechanism between SLL GS/GT and SLL GT/GS accounts for their different phase change properties, with the thermal stability of GT/GS being higher than that of GS/GT. These significant findings lay a solid foundation for the design of novel PCM structures with superlattice-like configurations, facilitating the exploration of advanced PCMs with enhanced thermal properties.

## 4. Conclusions

This paper presents a systematic investigation of the phase transition properties of SLL GS/GT and SLL GT/GS under different sputtering sequences. The *T_ten_* values of SLL GS/GT and SLL GT/GS are measured to be 124.3 °C and 151.9 °C, respectively, both significantly higher than that of GST (85 °C). Moreover, the crystallization activation energies of SLL GS/GT and SLL GT/GS are determined to be 2.4 eV and 2.6 eV, further highlighting their favorable thermal stability. Notably, the thermal stability of SLL GT/GS is found to be superior to that of SLL GS/GT. This difference in thermal stability mainly arises from the distinct atomic behavior induced by different sputtering sequences. When the GS layer is positioned below, the presence of the Sb precipitated phase in GS promotes the crystallization of GT, resulting in lower thermal stability for SLL GS/GT. In contrast, when GT is placed as the lower layer, the Sb precipitated phase formed in GS diffuses to GT, which reduces its impact on the thermal stability of the thin film, leading to an increase in the thermal stability for SLL GT/GS. In conclusion, the choice of the sputtering sequence exerts a significant influence on the properties of superlattice-like PCMs. These findings lay a strong foundation for the exploration of new PCM materials with enhanced performance.

## Data Availability

Data are contained within the article and Supplementary Materials.

## Supplementary Materials

The following supporting information can be downloaded at: https://www.mdpi.com/article/10.3390/ma17112773/s1, Figure S1: Temperature-dependent sheet resistance curves of SLL-GS-GT with different periods at a heating rate of 10°C/min. (a) [Ge_8_Sb_92_(25nm)-GeTe(25nm)]_1_ and [GeTe(25nm)-Ge_8_Sb_92_(25nm)]_1_ with single period; (b) [Ge_8_Sb_92_(6nm)-GeTe(6nm)]_4_ and [GeTe(6nm)-Ge_8_Sb_92_(6nm)]_4_ with four periods; (c) [Ge_8_Sb_92_(5nm)-GeTe(5nm)]_5_ and [GeTe(5nm)-Ge_8_Sb_92_(5nm)]_5_ with five periods. Reference [35] is cited in the Supplementary Materials.
