# Supplementary material for: The Effect of Sputtering Sequence Engineering in Superlattice-like Sb-Rich-Based Phase Change Materials
Source: Materials (Basel). 2024 Jun 6;17(11):2773. doi: 10.3390/ma17112773 (PMC11173929; doi:10.3390/ma17112773)
Supplement: Supplementary file 1 [file materials-17-02773-s001.zip › materials-2976338-supplementary.pdf]

Supporting Information:

The Effect of Sputtering Sequence Engineering in Superlattice-like Sb-Rich-Based Phase Change Materials

Anding Li [1], Ruirui Liu [1,*], Liu Liu [1], Yukun Chen [1] and Xiao Zhou [2,*]

1 School of Materials Science and Engineering, Shanghai Institute of Technology, Shanghai 201418, China; a2476157229@163.com (A.L.); liuliu020623@163.com (L.L.); growl_joke@163.com (Y.C.)

2 State Key Lab of Metal Matrix Composites, School of Materials Science and Engineering, Shanghai Jiao Tong University, Shanghai 200240, China.

* Correspondence: lrrsanxincao@sit.edu.cn (R.L.); zhouxiao113@sjtu.edu.cn (X.Z.)

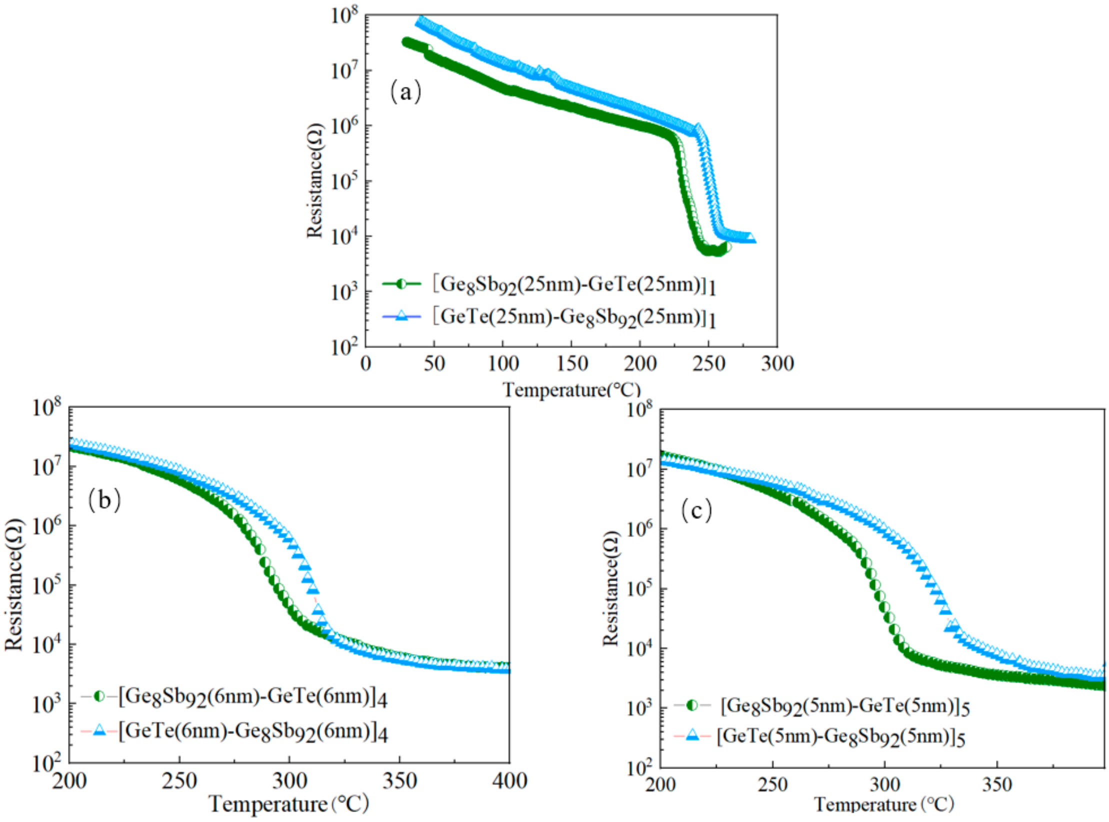

Figure S1. Temperature-dependent sheet resistance curves of SLL-GS-GT with different periods at a heating rate of 10° C / min. (a) $[Ge_8Sb_{92}$ (25nm)-GeTe (25nm)$]_1$ and [GeTe (25nm)- Ge_8Sb_{92} (25nm)$]_1$with single period; (b) $[Ge_8Sb_{92}$ (6nm)-GeTe (6nm)$]_4$ and [GeTe (6nm)- Ge_8Sb_{92} (6nm)$]_4$ with four periods; (c) $[Ge_8Sb_{92}$ (5nm)-GeTe (5nm)$]_5$ and [GeTe (5nm)- Ge_8Sb_{92} (5nm)$]_5$ with five periods.

Figure S1 shows the temperature-dependent sheet resistance curves of SLL-Ge_8Sb_{92}-GeTe with varying periods, obtained at a heating rate of 10° C / min. Evidently, all the SLL-Ge_8Sb_{92}-GeTe configurations exhibit a consistent pattern of change. Especially, the [GeTe (X nm)-Ge_8Sb_{92} (X nm)]$_y$ configuration demonstrates greater thermal stability compared to the [Ge_8Sb_{92} (X nm)- GeTe (X nm)]$_y$ arrangement, where X represents the thickness of the thin films and y indicates the number of periods. A detailed analysis of this aspect is provided in the main text.

Moreover, the multi-period SLL-Ge_8Sb_{92}-GeTe exhibits a higher crystalline temperature compared to the single-period configuration, primarily because of the restraining influence of multiple interfaces. This effect narrows the phase transition region of the phase change material and hinders its crystallization process, consequently bolstering its thermal stability [S1]. The phenomenon of significant temperature variation during crystallization under different time periods will undoubtedly be a focal point in our exploration of phase change materials.

In the interest of experimental convenience, the main text predominantly concentrates on the single-period thin films, specifically [Ge_8Sb_{92} (25 nm)-GeTe (25 nm)]$_1$ and [GeTe (25 nm)-Ge_8Sb_{92} (25 nm)]$_1$.

Reference

[S1]. Raoux, S.; Cheng, H.-Y.; Jordan-Sweet, J. L.; Munoz, B.; Hitzbleck, M. Influence of interfaces and doping on the crystallization temperature of Ge–Sb. *Appl. Phys. Lett.* **2009**, *94*, 183114.
